# From temporary event spaces to permanent urban communities: A physical-relational-legacy framework for Olympic Village transformation

**DOI:** 10.1371/journal.pone.0359591

**Published:** 2026-10-06

**Authors:** Yezi Liu, Shengchen Zhang, Hai Li

**Affiliations:** 1 School of Economics and Management, Shanghai University of Sport, Shanghai, China; 2 China Football College, Beijing Sport University, Beijing, China; Tsinghua University, CHINA

## Abstract

The Olympic Village has evolved over time to become a full-fledged urban system that encompasses not just the temporary housing needs of athletes participating in the Olympic Games but also infrastructure development, governance coordination, sustainability planning, and long-term community transformation. Although there are many studies of Olympic Villages, they typically focus on a single aspect of them such as the physical redevelopment of an area, the way that it was governed during the Olympics, and what happened to the Village after the Games ended. Existing studies have examined various dimensions separately, but fewer studies integrate these dimensions into a unified lifecycle framework. To fill this gap, this study proposed the Physical-Relational-Legacy (P-R-L) framework to help explain the changes in form that occur in Olympic Villages over their lifecycle. Using a Straussian grounded theory approach and comparative case study methodology, the study investigated six Olympic events: the Summer Olympics of 2008, 2012, 2016, 2020, and 2024, and the Winter Olympics of 2022. The data collected for this study included official Olympic reports, operational manuals, planning documents, and other types of published materials related to legacy. Using systematic open, axial, and selective coding supported by constant comparison, the study inductively developed the three interrelated dimensions: Physical, Relational, and Legacy. The results of this study indicated that the transformation of an Olympic Village is not simply a matter of developing some areas after the games end. Rather, it involves a series of interactions between changing infrastructure systems, governance mechanisms, and ongoing legacy development processes. Moreover, each of these dimensions interacts with one another at all stages of an Olympic Village’s lifecycle instead of occurring sequentially. The study contributes by providing an integrated analytical framework explaining how physical adaptation, governance coordination, and legacy formation interact throughout the Olympic Village lifecycle.

## 1 Introduction

Mega-sport events like the Olympics provide increasing opportunities for urban transformation, experimental sustainability, and innovative governance. One of the key physical and institutional infrastructures that accompany large-scale sport events is the Olympic Village. The Olympic Village serves a dual function, operating both as a temporary event time-based housing system for athletes and officials and as a longer-term urban development strategy. For over twenty years, there has been a shift in the planning rationale of Olympic Villages away from being solely focused on providing temporary residential accommodation toward broader objectives of integrating into sustainable urban environments, promoting innovative forms of governance, and creating a legacy [1]. In this context, Olympic Villages are increasingly viewed not simply as temporary residential facilities but rather as integrated urban systems that support infrastructure modernization, facilitate community transition, and ultimately lead to post-Games redevelopment [2,3].

Despite the ambitious goals outlined above, the transition from temporary sports infrastructure to a sustainable permanent community remains fraught with difficulty. Post-Games challenges associated with Olympic Villages include unused or poorly utilized sports infrastructure, poor integration of the Olympic community into local communities, financial burden, disintegrated governance models, and, in some cases, the emergence of “white elephants” where planned long-term community functions fail to materialize [4–6]. The fundamental conflict existing between the temporary nature of the Olympic Village’s operational use during the Games versus its required longevity as an urban entity post-Games represents a major challenge within mega event governance/urban sustainability research [7]. Although some examples of Olympic Villages have demonstrated success in achieving post-Games urban regeneration outcomes, such as London 2012, other Olympic Villages have failed to create enduring social and economic viability illustrating considerable variability in legacy creation across different institutional and urban contexts [8]. Previous studies have analyzed the Olympic Village with the perspectives of architecture, construction sustainability, urban development and revitalization, user experience, and legacy planning for the Village [9–12]. Research has also documented the increasing importance of governance coordination, resilience planning, and community-based developments within the context of mega-event infrastructure projects [13,14]. However, the existing literature remains fragmented in at least two important respects. Firstly, previous studies usually focus on physical infrastructure, governance systems, social interaction, or legacy outcomes as largely separate domains [15]. In doing so, they fail to adequately account for the dynamic interactions across these respective dimensions throughout the Olympic Village lifecycle. Secondly, most studies view Olympic Village transformation as a linear progression from temporary use during the Games to permanent use after the Games [16]. Such approaches inadequately explain how temporary and permanent operational logics coexist and influence one another across multiple stages of planning, operation, and long-term urban transition. In practice, the Olympic Village simultaneously accommodates multiple operational logics throughout its lifecycle, including event-time security management, long-term residential planning, commercial redevelopment, social integration, and governance transfer. There remains a need for an integrated analytical framework that can explain how these interrelated dimensions collectively influence the long-term transformation of Olympic Villages into sustainable urban communities [17].

In response to these gaps, this study conceptualizes the Olympic Village as a dynamic temporary community structure characterized by continuous interaction among physical infrastructure, relational governance systems, and long-term legacy formation. Drawing on grounded theory and cross-case documentary analysis, this study examines six Olympic events to inductively identify recurring patterns of transformation across diverse governance and urban contexts. This methodological approach is particularly appropriate because existing theory does not adequately explain how physical, governance, and legacy processes interact throughout the Olympic Village lifecycle. It proposes a Physical-Relational-Legacy (P-R-L) model to explain the mechanisms through which Olympic Villages achieve structural transition from temporary exceptional spaces to permanent urban communities. The analysis draws upon a comparative documentary corpus consisting of official Olympic reports, governance documents, sustainability plans, operational manuals, and media reports collected across the six Olympic cases. The study focuses particularly on how adaptive infrastructure, governance translation, and legacy accumulation interact dynamically throughout the Olympic Village lifecycle.

The study contributes to the literature in three ways. First, it proposes the P-R-L framework for examining Olympic Village transformation across multiple lifecycle stages. Rather than treating infrastructure, governance, and legacy as separate domains, the framework emphasizes their dynamic relationships throughout planning, Games-time operation, and post-Games redevelopment. Second, the study expands current discussions of Olympic legacy by highlighting the recursive interactions among physical adaptation, governance coordination, and long-term community transformation. Third, the findings outline a lifecycle-oriented governance model for policymakers and urban planners in Olympic Village design that effectively support sustainable urban development, governance continuity, and urban integration. In this respect, the framework aligns with broader sustainability objectives reflected in SDG 11 (Sustainable Cities and Communities) and SDG 16 (Peace, Justice and Strong Institutions).

To methodologically address the identified research gaps, this research utilizes a grounded theory approach coupled with comparative multi-case methodology that has been applied to six Olympics held across various governance and urban contexts. In addition to providing the P-R-L framework for explaining the interrelationship among adaptive infrastructure systems, governance coordination mechanisms, and long-term legacy formation, the data collected in this study is analyzed using iterative coding and cross-case comparisons. This study pursues three objectives: a) to examine the structural transformation of Olympic Villages across different stages of their lifecycle, b) to identify the interactions among physical infrastructure, relational governance, and legacy formation through grounded theory analysis of different Olympic cases, and c) to propose an integrated analytical framework that explains these interactions across diverse governance and urban contexts. The overall research design structure used in this study is illustrated in Fig 1 below.

**Fig 1:**
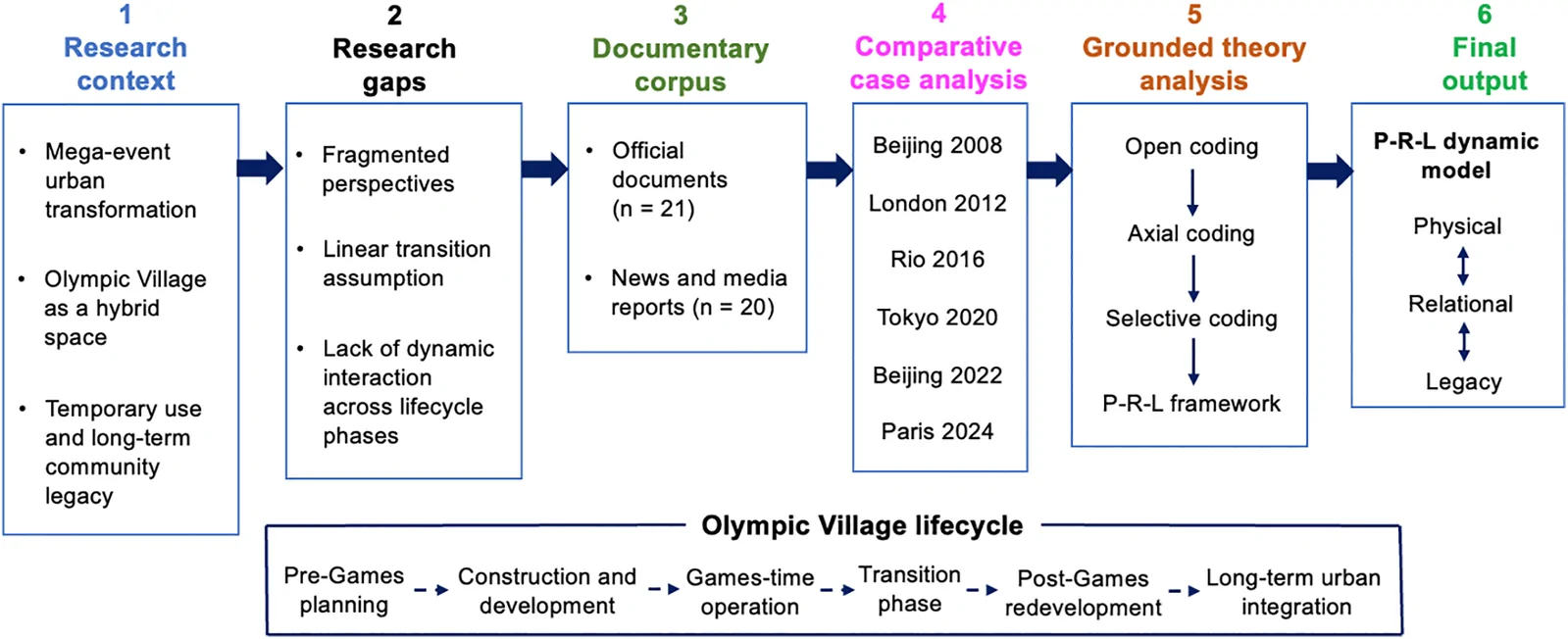
Integrated research framework for Olympic Village transformation analysis.

## 2 Literature review

### 2.1 Olympic Villages as temporary urban systems

The temporary housing arrangements provided at the Olympics, specifically the Olympic Village, have become an integrated part of a larger urban governance and development model. Originally conceived as a temporary residence for athletes, Olympic Villages are now increasingly planned as catalysts for urban regeneration, infrastructure modernization, sustainable development, and long-term post-Games legacy formation [18,19]. This growth shows a stronger connection being made between mega-events such as the Olympics and longer-term urban development plans. As cities seek to validate the massive financial investment, they make in staging mega events (such as the Olympics), there is increasing emphasis placed upon connecting mega-event planning to long-term urban development. Existing research has identified three key features of Olympic Villages as temporary urban systems. Firstly, Olympic Villages are some of the most concentrated forms of temporary space organization globally. They require extremely rapid deployment of residential environments which can house thousands of athletes, officials, and other personnel involved in supporting the Games over a short timeframe [20,21]. The extreme time constraints under which Olympic Villages are developed distinguish them from traditional urban developments, where time pressures are much less pronounced and requires highly coordinated and specialized governance and infrastructure systems [22]. Secondly, scholars are highlighting the need for Olympic Villages to include flexible and adaptable physical infrastructure. Research on modular buildings, prefabricated structures, and reversible spatial designs are demonstrating how Olympic Villages serve as testbeds for sustainable urban construction techniques [23]. The ability to adapt spatial configurations of Olympic Villages after the Games will determine whether costs associated with demolition can be minimized and environmental waste reduced [24,25]. Therefore, Olympic Villages can be viewed as transitional urban spaces that provide for both temporary uses during the Games and long-term uses in their new form. Thirdly, Olympic Villages are socially complex temporary communities that are culturally diverse, densely populated with people interacting with each other intensely, and subject to extraordinary levels of governance control [26]. Previous studies have looked at various aspects of the athlete experience including their intercultural interactions, psychological adaptations, and the creation of “the Olympic family” identity in village settings [27,28]. There is also evidence that social inclusion mechanisms, common public spaces, and multicultural services contribute to forming temporary sense of community among residents of Olympic Villages during the Games [29,30].

Although these studies have substantially advanced understanding of Olympic Villages, they have largely examined physical infrastructure, governance arrangements, and community dynamics as separate analytical domains. Consequently, the literature provides limited insight into the dynamic relationship of these dimensions throughout the entire lifecycle of an Olympic Village. This fragmentation makes it difficult to explain how decisions made during one phase influence governance, community development, and legacy outcomes in subsequent stages. Therefore, a more integrated analytical perspective is needed to capture the interdependence of physical, relational, and long-term transformation processes.

### 2.2 Mega-event legacy and urban transition

Legacy is an increasingly important part of modern mega-event research, especially after growing concerns about sustainable Olympic infrastructure investments were expressed [31]. Research on Olympic legacy usually assesses if/and/or to what extent the hosting city can develop lasting (economic, social, environmental and institutional) benefits by using a mega-event as a platform to create those benefits after the Games are completed [32]. Thus, the use of Olympic Village(s), one of the few types of Olympic infrastructure developed for post-Games urban reuse, is a strategic part of that process. There is a large amount of research documenting the impact of Olympic Villages in creating new uses of abandoned urban spaces through urban regeneration and changing the physical landscape. For example, studies of former Olympic hosts (London, Barcelona, Beijing, and Rio De Janeiro) have documented ways in which the Olympic Village has been used to spur development of areas with declining populations and businesses through improved transit systems and increased housing stock, and to create interest from private investors in these areas long after the Olympics concluded [33]. In many cases, former Olympic Villages have been converted into residential communities, multi-use developments, educational facilities, and business centers [34]. However, physical redevelopment alone does not necessarily constitute a successful legacy as long-term outcomes also depend on factors including residential affordability. Community integration, governance continuity, and sustained social and economic vitality.

Legacy outcomes vary considerably across host cities. While many of the Olympic Villages have been very successful in creating positive urban transformation for their respective cities, several of them have experienced severe issues post-Games, such as vacant buildings, low levels of social interaction among residents, large-scale speculation in real estate, and poor financial performance [35]. More recently, research indicates that the physical redevelopment strategies of an Olympic Village as well as important factors like governance coordination, institutional continuity, and long-term community integration determine its overall success. In addition, there is growing interest in expanding our definitions of what constitutes Olympic legacy beyond just economic/infrastructure measures. Recent studies place greater emphasis on less tangible types of legacy, including increased governance capacity, institutional learning, resilience development, and increased social capital [36]. After the COVID-19 pandemic and subsequent challenges surrounding the organization of the 2020 Summer Olympics, researchers have shown a great deal of interest in identifying how mega events can contribute to urban resilience and emergency governance capabilities [37]. These new trends suggest that Olympic legacy is a multi-faceted and dynamic process rather than a fixed post-event outcome. These observations suggest that Olympic legacy should be understood as an evolving process shaped by the interaction of infrastructure adaptation, governance arrangements, and community development, rather than solely as a post-event assessment of redevelopment outcomes.

### 2.3 Governance, resilience, and community transformation

The multi-actor environment associated with Olympic Village development is among the most significant issues facing planners. It is common for at least six different types of actors to interact over varying levels of spatial scale (national government, local municipality, Olympic organizing committee, private developer, community organization, and international governing body) and over several temporal phases [38,39]. These interactions increase in complexity when there are strict event deadlines, financial risk, and public scrutiny. Existing literature on mega-event governance emphasizes the need for effective forms of collaborative and adaptive governance. Multi-stakeholder coordination processes, public-private partnerships, and integrated project management frameworks have been identified as key components necessary to ensure successful project completion and operational performance [40]. However, research also indicates that governance fragmentation and conflicting stakeholder agendas continue to represent major barriers to achieving long-term legacies for mega events [41]. Resilience is an increasingly prominent theme in research examining mega-event governance. Urban resilience is defined as the ability of urban areas and their institutions to functionally recover, reorganize, or perform under stressful or uncertain conditions [42]. Due to their unique characteristics of extreme density of population, cultural diversity, security concerns, and uncertainty around operations, Olympic Villages represent particularly compelling contexts for assessing resilience [43]. Research has shown that emergency response systems and digital platforms for governance along with other forms of cross-agency coordination employed during mega events can serve to enhance broader capacities for urban governance after the event [44]. Within the present study, adaptive governance, urban resilience, and social capital are treated as theoretical perspectives that sensitize the analysis rather than as predetermined analytical categories. These perspectives provide conceptual guidance for interpreting documentary evidence while the final dimensions of the proposed P-R-L framework emerge inductively through the grounded theory coding process.

Social capital theory also offers valuable insight into the relational aspects of Olympic Village communities. “Bridging social capital” [45] formed through shared engagement in public settings and institutionalized trust contributes to cooperative behaviors among diverse social groups. Multicultural public spaces and services in addition to community-building activities conducted during the Olympics could contribute to forming meaningful, though often short-lived, social bonds among individuals drawn from extremely heterogeneous populations [46]. These relational dynamics may impact the extent to which post-Games communities will integrate socially over the long run. Although governance, resilience, and social integration have each been examined extensively within previous mega-event research, relatively few studies have investigated how these processes interact across the full lifecycle of Olympic Villages. This limitation highlights the need for an analytical framework capable of explaining how physical infrastructure, governance relationships, and legacy formation co-evolve over time.

### 2.4 Research gaps and conceptual positioning

The preceding review identifies three principal limitations in the existing literature. First, research has largely examined infrastructure, governance, community dynamics, and legacy as separate analytical domains, providing only limited understanding of their interrelationships. Second, Olympic Village transformation is frequently conceptualized as a linear progression from temporary event use to permanent urban reuse, overlooking the coexistence and interaction of temporary and permanent operational logic. Third, relatively little research explains the mechanisms through which these interaction processes shape the long-term evolution of Olympic Villages across different governance contexts. In response to these limitations, this study adopts a grounded theory approach to examine Olympic Villages as evolving temporary community systems defined by continuous interactions among adaptive infrastructure, relational governance, and legacy formation. Rather than testing predetermined theoretical relationships, this study seeks to develop an integrated analytical framework that explains how these dimensions emerge and interact across multiple Olympic cases. Table 1 summarizes the major research streams, their limitations, and the positioning of the present study.

**Table 1:** Literature gaps and conceptual positioning of the present study.

Research stream	Existing focus	Key limitation	Contribution of present study
Olympic Village infrastructure studies	Physical redevelopment, architecture, sustainability design	Physical systems often examined independently from governance and legacy processes	Examines infrastructure adaptation within a dynamic socio-spatial transformation framework
Mega-event governance research	Coordination mechanisms, institutional management, public-private partnerships	Limited attention to lifecycle interaction between governance and urban transition	Provides an analytical perspective on how governance systems evolve across temporary and permanent operational phases
Olympic legacy studies	Economic outcomes, urban regeneration, post-Games reuse	Legacy frequently conceptualized as a linear post-event outcome	Conceptualizes legacy as a recursive and evolving process throughout the lifecycle
Temporary community and social integration studies	Athlete interaction, multicultural environments, social cohesion	Weak integration with infrastructural and governance dimensions	Connects relational dynamics with infrastructure adaptation and legacy formation
Urban resilience and sustainability research	Crisis management, adaptive governance, smart-city systems	Resilience often analyzed separately from Olympic Village transformation	Incorporates resilience accumulation and institutional continuity into the P-R-L framework
Present study	Dynamic Olympic Village transformation	Addresses fragmented and linear approaches in prior literature	Proposes the P-R-L model through grounded theory and comparative multi-case analysis

## 3 Research methodology

### 3.1 Research design

The current study adopted a grounded theory approach, informed by [47] to examine the structural evolution of Olympic Villages across different lifecycle stages. This approach was selected because existing studies provide valuable but fragmented explanations of Olympic Village transformation while an integrated understanding of the interactions among physical infrastructure, governance, and legacy processes remains underdeveloped [48,49]. Furthermore, Olympic Village transformation involves interactions among physical infrastructure, governance arrangements, temporary communities formed during Olympics, and post-Games long-term legacies. Understanding the interactions of these dimensions requires an analytical approach that can capture complex and evolving relationships across different stages of the Olympic Village lifecycle. A grounded theory approach is particularly appropriate because it enables the inductive identification of relationships and processes from empirical evidence, making it suitable to examine how Olympic Villages evolve from temporary event spaces into permanent urban communities. Therefore, the study follows the Straussian grounded theory procedures of open, axial, and selective coding to develop an empirically grounded analytical framework from documentary evidence.

In contrast to deductive approaches based on pre-defined hypotheses, grounded theory is concerned with developing theoretically informed conceptualizations based upon empirical evidence. Thus, this study developed a single conceptual model to explain how Olympic Villages evolve over time. Following the Straussian grounded theory procedures of open coding, axial coding, and selective coding, concepts and their relationships were successively identified and clarified throughout the course of the research. Grounded theory was particularly suited to this study because the transformation of Olympic Villages represents multiple dimensions of interactions between spatial infrastructure, governance systems, temporary community dynamics, and urban legacy formation, all occurring within distinct institutional and temporal environments.

### 3.2 Case selection and data collection

The study utilized a comparative multi-case study approach to examine six Olympic events including the Summer Olympics of 2008, 2012, 2016, 2020, and 2024 and the Winter Olympics of 2022. Because the long-term legacy of Paris 2024 is still evolving, the analysis focuses on documented planning strategies, governance arrangements, sustainability objectives, and early redevelopment initiatives rather than fully realized post-Games legacy outcomes. These cases were purposively selected as they represent information-rich examples of different governance models, urban regeneration strategies, sustainability agendas, and legacy trajectories. The number of Olympic Village cases has been determined as adequate for theoretical saturation based upon repeated comparisons of these cases that resulted in consistent conceptual relationships and no additional significant structural dimensions beyond those initially identified through earlier stages of coding and data analysis. Cross-case comparisons among the selected Olympic Village cases have increased the opportunity for analytical generalizations. Specifically, this study chose to capture key changes in Olympic governance that include adaptable infrastructure planning, sustainable development, resilient governance, smart-city integration, and post-COVID operational management.

Documentary analysis was used as the primary method of collecting data. The core documents were collected from official Olympic reports, operational manuals, bid documents, legacy reports, and planning materials for the Olympic Villages. Supporting documentation included sustainability reports, governance documents, and news articles published by internationally recognized and reputable media organizations. Media sources were used only as supplementary evidence to contextualize official documentary materials and to triangulate governance developments and public reporting. Documentary material provided an abundance of information about the construction of infrastructure, coordinating governance activities, organizing services, transforming space, and developing areas after the Games. A total of 41 core documents were screened systematically and formed the basis of a comprehensive corpus. This set of core documents consisted of official reports, operational guides, planning materials, and publications related to legacy. The documentary dataset (Table 2 in S1 File) provided in-depth evidence related to how Olympic Villages were planned, operated, and governed in a variety of urban settings.

**Table 2:** Documentary corpus and analytical purpose.

Source type	Number of documents	Representative materials	Primary analytical purpose
Official Olympic reports	8	IOC evaluation reports, official Olympic reports	Infrastructure planning and governance systems
Legacy and sustainability reports	5	Legacy strategy reports, sustainability assessments	Long-term urban integration and legacy outcomes
Operational manuals and governance documents	8	Host city operational manuals, governance frameworks	Games-time coordination and institutional management
News and media reports	20	International and local media coverage	Public narratives, governance interpretation, operational dynamics
Total documentary corpus	41	Multi-source documentary dataset across six Olympic cases	Comparative grounded theory analysis

Because official planning and legacy documents may reflect institutional perspectives, findings were interpreted through triangulation across multiple documentary sources, including governance reports, sustainability documents, operational manuals, and independent media coverage, thereby reducing reliance on any single institutional narrative. Table 3 summarizes the major characteristics and transformation patterns observed across the selected Olympic events.

**Table 3:** Comparative overview of Olympic Village cases and transformation strategies.

Olympic event	Physical strategy	Governance logic	Legacy outcome	Key post-Games transformation	Distinctive feature	Primary documentary sources	Representative coding themes
2008 Summer Olympics (Beijing)	Large-scale new construction; integrated urban expansion; permanent residential planning	Strong state-led coordination and centralized multi-agency governance	Urban integration and long-term residential redevelopment	Conversion into residential districts and urban expansion zones	“Green Olympics” and large-scale urban modernization	IOC reports; Beijing Olympic planning reports; sustainability reports; media coverage	Green modernization; centralized coordination; urban expansion; infrastructure-led transformation
2012 Summer Olympics (London)	Modular and flexible housing systems; sustainable infrastructure design	Public–private partnership (PPP) governance with community-oriented planning	Social housing regeneration and community continuity	Transformation into East Village residential community	Legacy-first planning and sustainability-centered regeneration	Legacy reports; governance frameworks; sustainability plans; operational manuals	Legacy-first planning; modular infrastructure; social housing integration; community continuity
2016 Summer Olympics (Rio de Janeiro)	New construction combined with partial venue reuse and clustered urban design	Multi-level governance with market-oriented redevelopment strategy	Commercial and residential redevelopment with uneven community integration, housing affordability challenges, and mixed long-term social outcomes	Conversion into mixed-use commercial and residential districts	Infrastructure-led urban revitalization	Urban redevelopment plans; governance reports; media reports; operational documents	Market-oriented redevelopment; governance fragmentation; mixed-use conversion; uneven legacy realization
2020 Summer Olympics (Tokyo)	High-density smart infrastructure with temporary-to-permanent conversion capacity	Centralized governance emphasizing emergency management and operational resilience	Mixed-use urban district and governance resilience legacy	Transformation into Harumi residential and commercial district	Pandemic-driven governance adaptation and resilience management	COVID-19 operational manuals; governance reports; legacy reports; media coverage	Emergency governance; resilience adaptation; smart infrastructure; closed-loop management
2022 Winter Olympics (Beijing Winter Olympics)	Low-impact mountain cluster development and smart infrastructure systems	Digital governance and sustainability-oriented regional coordination	Talent housing, tourism development, and regional integration	Long-term reuse for tourism, training, and residential functions	Smart governance and ecological sustainability integration	Sustainability reports; smart city planning documents; Winter Olympics reports; media reports	Ecological sustainability; digital governance; tourism integration; resilience planning
2024 Summer Olympics (Paris)	Existing venue utilization, low-carbon design, and circular economy planning	Inclusive governance emphasizing climate responsibility and stakeholder collaboration	Early legacy implementation emphasizing a low-carbon urban district and resilient community development	Transformation into mixed-use sustainable urban neighborhood	Climate-conscious Olympic governance and social inclusiveness	Paris 2024 sustainability strategy; governance documents; legacy planning reports; media narratives	Circular urbanism; low-carbon transition; inclusive governance; sustainable community integration

### 3.3 Data coding and analytical procedures

The coding process followed the grounded theory principle of constant comparison, whereby concepts and categories were continuously refined through iterative comparison across documents and cases [47]. The documentary materials were coded through iterative reading, constant comparison, and repeated refinement of concepts and categories. Coding decisions and category development were regularly reviewed through analytical discussions to improve conceptual consistency and methodological rigor. The analysis proceeded through three sequential stages: open coding, axial coding, and selective coding. During open coding, representative statements related to Olympic Village planning, governance, operation, and legacy transformation were extracted from the documentary corpus and decomposed into discrete conceptual units. Through repeated comparison and abstraction, 326 open codes were identified from empirical materials. These open codes were subsequently consolidated into 90 initial concepts representing recurring structural characteristics within Olympic Village transformation processes. The second stage involved axial coding through which conceptually related ideas were reorganized into higher-order analytical categories. Through continuous comparison across cases, the 90 concepts were consolidated into 24 axial categories and further grouped into seven major categories. These categories comprised adaptive infrastructure, collaborative delivery systems, governance coordination, service network activation, civic spatial transition, industrial value amplification, and institutional resilience accumulation, which were subsequently integrated into three interconnected structural dimensions: Physical, Relational, and Legacy. The Physical dimension primarily concerned adaptive infrastructure and collaborative delivery systems. The Relational dimension focused on governance coordination, service systems, and community interaction. The Legacy dimension captured long-term urban integration, economic redevelopment, and institutional resilience outcomes.

Selective coding constituted the final stage of analysis and focused on integrating the major categories into a unified explanatory framework. Through repeated theoretical comparison, the core analytical phenomenon was identified as the dynamic transition of Olympic Villages from temporary event spaces into permanent urban communities through interacting infrastructural, governance, and legacy mechanisms. This process ultimately resulted in the development of the P-R-L model. Fig 2 illustrates the grounded theory coding and theoretical integration process adopted in this study including data collection, open coding, conceptual abstraction, axial coding, selective coding, and final P-R-L model development.

**Fig 2:**
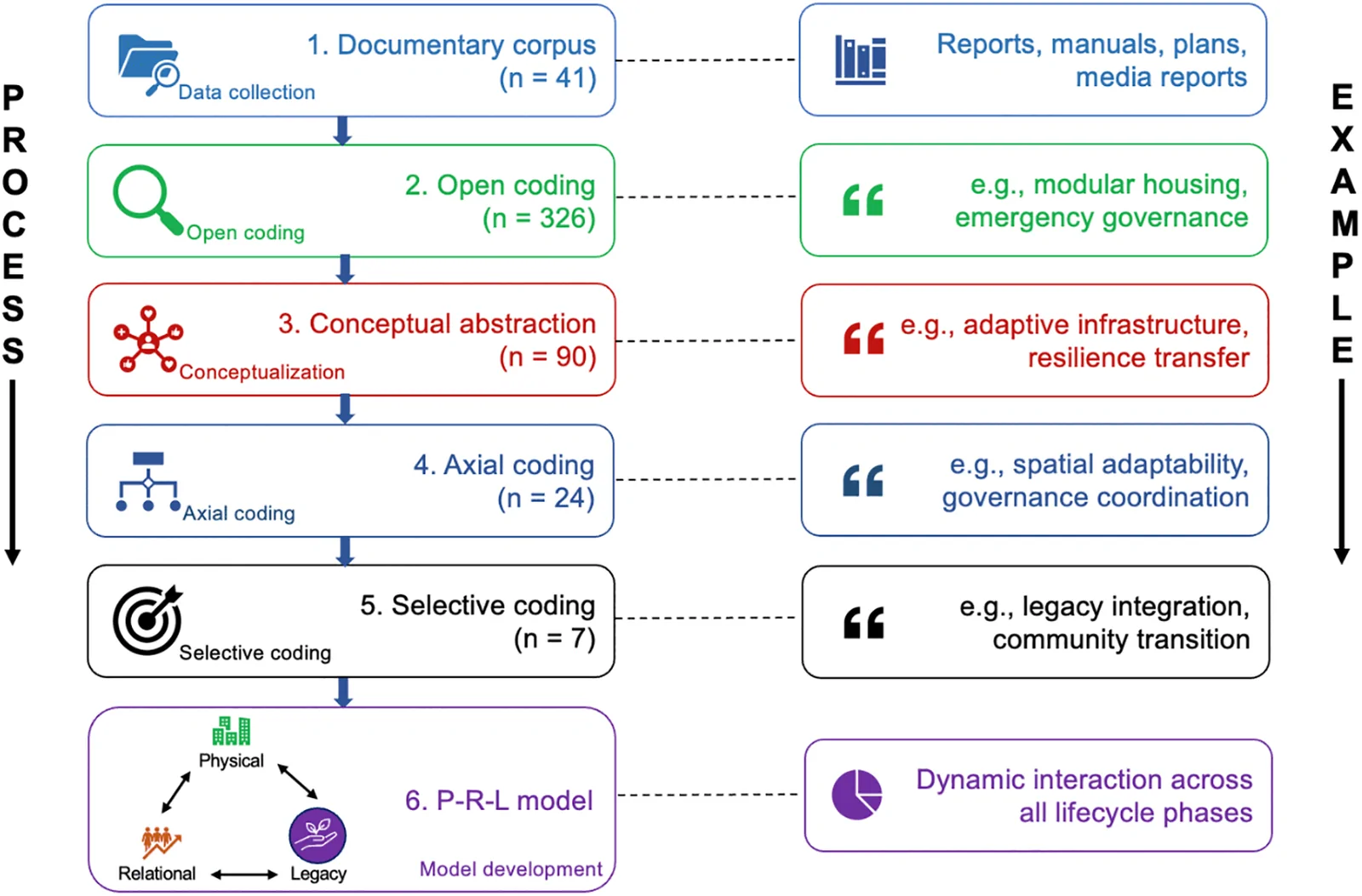
Grounded theory coding and P-R-L model construction.

Table 4 (in S2 File) presents representative examples illustrating the progression from documentary extracts through open coding, conceptual abstraction, axial coding, and final integration into the P-R-L framework. Owing to journal space limitations, only representative examples are presented.

**Table 4:** Example of grounded theory coding process.

Representative analytical extract	Original expression	Documentary source	Olympic case	Open code	Initial concepts	Axial category	Core dimension
“Flexible housing units designed for post-Games conversion”	“Success lies in holding both the Games setup and the post-Games conversion in mind from the start and throughout all design choices. London 2012 offers a benchmark. The East Village in Stratford – initially housing 17,000 athletes – transitioned into more than 2,800 new homes, including affordable housing.”	Wtpartnership (https://wtpartnership.com.au/insights/getting-accommodation-right-from-games-to-legacy/)	London 2012	Modular housing	Adaptive infrastructure	Spatial adaptability	Physical
“Closed-loop operational management during pandemic conditions”	“Learning from the experience and lessons of the Tokyo Olympics, Beijing has applied a strict closed-loop management policy, which is unprecedented in a major comprehensive global sporting event. ”	Peopledaily (https://peoplesdaily.pdnews.cn/bri-news/er/30001413793)	Beijing 2022	Emergency coordination	Governance flexibility	Adaptive governance	Relational
“Post-Games conversion of the Olympic Village into residential and mixed-use functions”	“After the Paralympics concluded, designers transformed the Olympic Village into a mixed-use development with 82 buildings housing 6,000 inhabitants and offices for another 6,000 workers.”	Parametric architecture (https://parametric-architecture.com/olympic-village-across-10-cities/?srsltid=AfmBOor_9yjPTGEZSeRTJdfIda1X5hxqZOLsmvxfDPHaTNf-ft8LDvSd)	Paris 2024	Residential transition	Urban integration	Civic spatial transition	Legacy
“Digital governance platforms reused after the Games”	“Atos’ Urban Data Platform provides us with innovative digital infrastructure to meet our targets to decarbonize the Athletes’ Village and make sustainable investments in technology” commented Antoine Du Souich, Strategy and Innovation Director, SOLIDEO.“We are proud that SOLIDEO has decided to include Urban Data Platform in the delivered works since they herald Paris as the city of tomorrow. Urban Data Platform reflects our capabilities to support cities and territories in the management of the data lifecycle. Leveraging our expertise in cybersecurity and the sovereign cloud, UDP converts raw data into tools that monitor decarbonization policies, urban planning, crisis management and quality of life for contemporary urban spaces” said Yannick Tricaud, EVP, CEO Southern Europe, Atos.	Atos (https://atos.net/en/2022/press-release_2022_10_04/atos-wins-a-contract-with-solideo-olympic-delivery-authority-to-digitally-manage-the-athletes-village-with-its-smart-territories-data-platform-urban-data-platform)	Paris 2024	Smart governance reuse	Institutional continuity	Resilience accumulation	Legacy

The coding process demonstrates the progressive abstraction from raw documentary evidence toward higher-order conceptual categories and ultimately the construction of the P-R-L framework. Through iterative theoretical refinement and cross-case comparison, the study identified the dynamic interaction mechanisms underlying Olympic Village transformation across multiple lifecycle phases.

### 3.4 Reliability and theoretical saturation

Several methods were used to increase the methodological rigor and analytical dependability of this research. Firstly, the research utilized multi-documentary sources so that the data could be triangulated, and the reliance on a single document type could be reduced. Secondly, using the same set of documents for six different Olympic Games strengthened the identification of recurring concepts. All 41 documents in the analytical corpus were reviewed by the authors as part of the coding and analytical process. The reviewed material encompassed all document types represented in the corpus, including official Olympic reports, legacy and sustainability reports, operational and governance documents, and media reports, as well as all six Olympic cases examined in the study. The authors jointly examined the documentary evidence and coding outcomes throughout the analytical process to ensure consistency and methodological coherence. Following the initial coding process, two additional documents (in S1 File) not included in the core 41-document corpus were examined by the research team to assess whether new concepts, analytical categories, or relationships emerged. These comprised 1 official report of the Beijing 2008 Olympic Games and 1 operational guide of the Tokyo 2020 Olympic Games. Differences in interpretation were discussed until consensus was achieved. No new substantive concepts, category properties, or relationships emerged from the additional analyses, indicating that theoretical saturation had been achieved [50]. Therefore, the use of grounded theory allowed for the creation of an empirically based and theoretically integrative framework for describing the developmental trajectory of Olympic Village temporary community systems across various governance and urban settings.

## 4 Findings and discussion

### 4.1 Open coding and emergence of structural patterns

The first stage of analyzing grounded theory data involved open coding of the extensive collection of documentary data. By repeatedly reading the documents and constantly comparing them, many illustrative statements about how Olympic Villages are planned, governed, built, operated during the Games, and reused after the Olympics were systematically extracted. A total of 326 open codes were initially identified in the original dataset which was subsequently refined into 90 initial concepts through iterative conceptual abstraction. From this process it became clear that transforming an Olympic Village is much more than simply changing what is there physically and involves adapting the existing infrastructure to meet new needs, restructuring the governing processes surrounding that infrastructure, integrating those who have previously been excluded into a new urban community, and ultimately creating a lasting urban legacy.

Comparative analysis across the six Olympic Villages revealed recurring patterns and important contextual differences. London 2012 and Paris 2024 emphasized legacy-first planning and modular infrastructure, Beijing 2008 prioritized large-scale infrastructure-led urban expansion, Rio 2016 demonstrated market-oriented redevelopment with uneven social outcomes, Tokyo 2020 highlighted adaptive governance under pandemic conditions, and Beijing 2022 emphasized ecological sustainability and smart governance. Despite these contextual differences, common patterns of adaptive infrastructure, governance coordination, and long-term redevelopment emerged across the cases and informed the development of the P-R-L framework. The analysis demonstrates how modern Olympic Villages utilize reversible and adaptable spatial planning to support temporary accommodation during Games and long-term post-Games urban reuse. These utilities represent a trend towards planning the entire lifecycle of urban development rather than simply designing event spaces. The study further shows that the creation of an Olympic Village is typically accomplished under great duress resulting in high levels of time-sensitive collaboration between government agencies and private sector entities. Institutions involved in this process must work together through interagency coordination mechanisms, public-private partnership agreements, pre-Games readiness testing procedures, and risk-sharing contracts to create an Olympic Village within very aggressive timeframes. The Relational dimension defined concepts related to governance adaptation, emergency management, social interaction, and service network activation. For example, the closed-loop management systems used in the design and operation of Olympic Villages indicate that an Olympic Village is not just a mere residential accommodation, rather temporary communities governed with significant oversights and tremendous cultural diversity. As demonstrated by the governance adaptations employed at the 2020 Summer Olympics, an Olympic Village requires governance structures that can respond effectively to public health emergencies and address various types of operational uncertainty. The Legacy dimension explored the long-term impacts of a new urban development transformation after being previously developed as part of an Olympic Village. Recurring concepts identified through open coding included residential neighborhood conversion, governance handover, retail district growth, job creation, resilience knowledge transfer, and smart-city platform reuse. This study demonstrated that the legacy of Olympic Villages extends beyond the economic redevelopment of physical infrastructure, accumulating institutional learning, governance continuity, social inclusion, and urban resilience. The coding process further revealed that Olympic Village transformation should be viewed as a continuous interactive process shaped by long-term urban planning objectives and the evolving relationships among infrastructure, governance, and legacy development.

### 4.2 Axial coding and development of the P-R-L structure

The second phase of this analysis involved axial coding through which the 90 initial concepts were condensed down to 24 axial categories and subsequently grouped under seven major categories located on the three related structural dimensions (Physical, Relational, and Legacy). Several major categories fall within the Physical dimension, emphasizing adaptive spatial infrastructure and collaborative governance delivery mechanisms. Findings indicate that the Olympic Village’s physical space has shifted from being developed for temporary use to being adapted to remain functional post-Games. Examples of these include flexible zoning, modular partitions, adaptable utilities, and reversible construction techniques, all of which provide a means for spatial transformation to occur at an early point in the planning process. This adaptive infrastructure approach reflects a broader shift in Olympic urbanism from short-term spectacle-based development toward sustainable and long-term urban integration. Such developments have been implemented in 2012 and 2024 Summer Olympics where they employed front-loaded legacy planning and modular infrastructure approaches to facilitate rapid post-Games spatial conversions.

The Relational dimension measures the level of flexibility of governance and service network in Olympic Village communities. Findings indicated that the six examined Olympic Villages employed a dual-scenario governance model allowing for both Games-time exceptionality and post-Games community transition. During the Games, governance structures prioritized security management, operational coordination, emergency response, and athlete services. Following the Games, governance responsibilities progressively shifted toward municipal authorities, community organizations, and long-term urban management institutions, illustrating the transition from event governance to permanent community governance. Service networks and public interaction areas (e.g., shared dining spaces, cultural exchange spaces, digital service platforms, multilingual support services, etc.) are demonstrated as being the most important relational infrastructure to create temporary community cohesion. These findings support previous studies indicating that social infrastructure and bridging social capital contribute significantly to the development of temporary communities [51,52].

The Legacy dimension is comprised of civic spatial transition, industrial value amplification, and institutional resilience accumulation. The findings illustrate that a successful Olympic Village transformation depends upon not only the physical transformation of a space but also the incorporation of governance systems, public services, and economic use within pre-existing urban planning strategy. Successful examples include the redevelopment efforts of the Olympic Village after the London 2012 Games and the Beijing 2008 Games where Olympic Villages have been successfully converted into residential communities, educational institutions, and mixed-use urban districts. New forms of intangible legacies such as resilience knowledge transfers, digital governance systems, and long-term governance structures have also developed. However, the findings indicate varying degrees of success regarding legacy creation through Olympic Villages. While some Olympic Villages had higher levels of urban integration than others, many experienced problems associated with fragmented governance processes, speculative redevelopment, and lower long-term community vitality. These findings are consistent with previous criticisms indicating that Olympic legacy realization continues to remain heavily reliant on governance continuity and institutional coordination [53].

### 4.3 Dynamic evolution of Olympic Villages: The P-R-L model

Through selective coding and theoretical integration, this study identified a dynamic transition of Olympic Villages from temporary exceptional spaces to permanent urban communities via interacting physical, relational, and legacy mechanisms. Based on this central phenomenon, this study proposes the P-R-L model. The conceptual structure is illustrated in Fig 3.

**Fig 3:**
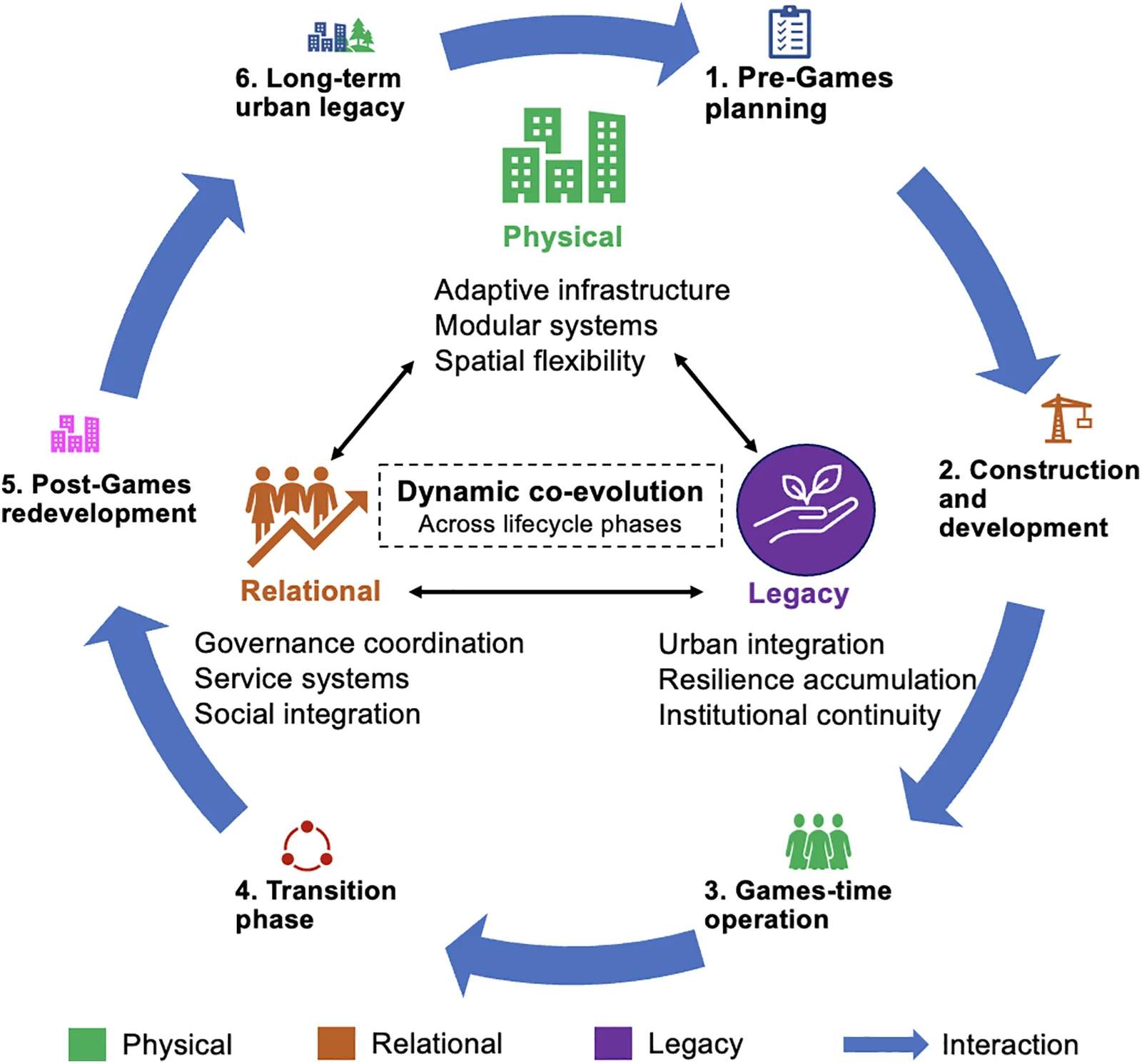
The P-R-L model.

The model envisions Olympic Village transformation as a dynamic and iterative process of ongoing interaction among adaptive infrastructure systems, governance translation mechanisms, and long-term urban legacy production. The findings demonstrate that the Physical dimension provides the material and organizational base for Olympic Village operation and transformation via modular infrastructure, adaptive utilities, and cooperative governance provision systems. The Relational dimension acts as the intermediate mechanism which enables governance adaptation and social integration by means of flexible institutional arrangements, emergency management systems, and service network activation. The Legacy dimension produces long-term urban values through spatial integration, economic redevelopment, governance continuity, and resilience accumulation.

The interaction among the three dimensions is recursive rather than sequential. Adaptive physical infrastructure enables particular forms of governance coordination by creating flexible spatial conditions for service delivery and community transition. Effective relational governance facilitates the implementation of long-term redevelopment strategies and institutional continuity, thereby strengthening legacy outcomes. In turn, anticipated legacy objectives influence infrastructure design and governance arrangements from the earliest planning stages. The strength of these interactions varies across governance contexts depending on institutional coordination, planning continuity, stakeholder collaboration, and redevelopment priorities. This continuous interplay of the three dimensions creates problems for traditional sequential models of Olympic legacy (pre-Games planning, Games-time operation, and post-Games use). The P-R-L framework does not support the view of an Olympic Village as a temporary events facility which will become a city district in time. It supports the view of Olympic Villages as constantly evolving systems of governance and space where temporary and permanent functions occur simultaneously during their entire existence.

The analysis further shows that successful transformation of an Olympic Village depends on the integration of spatial, relational, and institutional alignment. On the other hand, fragmented governance structures, limited institutional continuity, and narrowly commercial redevelopment strategies may result in uneven or less sustainable legacy outcomes.

### 4.4 Theoretical implications of dynamic Olympic Village transformation

The findings contribute to an expanding body of research in the field of mega-event urbanism, temporary communities, and Olympic legacy by developing a dynamic framework for analyzing the process of Olympic Village transformation. Olympic legacy research has commonly developed mega events through linear approaches to development which separate pre-Games planning, Games-time operations, and post-Games redevelopment into distinct temporal phases. The current study provides evidence that the transformation of an Olympic Village is characterized by the ongoing interactions between infrastructure systems, governance mechanisms, and long-term urban integration processes during its whole lifecycle. Hence, the proposed P-R-L framework extends previous Olympic legacy scholarship by conceptualizing Olympic Villages as recursively evolving social-spatial governance systems rather than as temporary event infrastructures transformed into urban districts after the conclusion of events. These findings suggest that successful Olympic Village transformation depends not simply on the presence of the three dimensions but on the strength of their interaction. Where infrastructure planning, governance coordination, and legacy objectives remain aligned throughout the lifecycle, long-term urban transformation is more likely to be achieved. Conversely, fragmented governance, discontinuous planning, or narrowly commercial redevelopment weakens these interaction mechanisms and contributes to uneven legacy outcomes.

The study also contributes to research on temporary urbanism and temporary communities. Previous research has predominantly studied Olympic Villages as transient living spaces used for athlete accommodations, cross-cultural engagement, and day-to-day management of events. However, the findings suggest that temporary Olympic communities can create long-lasting governance structures, institutional practices, and relational infrastructures that influence post-Games urban integration. This highlights the importance of temporary communities not as isolated events but transitional governance environments capable of promoting long-term changes in both urban and institutional terms.

The findings support the idea that emergency management systems, digital governance platforms, and adaptive operational coordination are key elements for creating functional Olympic Villages under conditions of uncertainty and crisis. Therefore, these results suggest that the concept of ‘resilience’ in mega-event governance is not only limited to a short-term crisis response capability (as seen with Tokyo 2020) or simply an ability to adapt to different types of crises (such as Beijing 2022), but rather it represents an ongoing process of institutional learning, governance adaptation, and long-term resilience accumulation both during and after the conclusion of the Games. The P-R-L model further provides new contributions to urban sustainability and lifecycle-oriented planning research by demonstrating the importance of integrating post-Games redevelopment objectives into early-stage infrastructure and governance planning. Thus, the successful transformation of an Olympic Village depends upon the compatibility and alignment of adaptive spatial infrastructure, governance continuity strategies, and long-term community integration strategies over multiple lifecycle phases. Therefore, this study is aligned with current arguments advocating lifecycle-oriented approaches to mega-event governance and sustainable urban development. Furthermore, this study contributes to contemporary discussions on adaptive urban governance by illustrating how large-scale temporary infrastructures can develop into long-term urban systems through coordinated interaction among spatial, relational and institutional transformation mechanisms. While developed from analyses of Olympic Villages, the P-R-L framework has potential analytical value for assessing other types of temporary urban systems such as international expos, festival cities, emergency settlements, and large-scale event-based urban redevelopment projects.

## 5 Conclusion

This study examined how Olympic Villages transform from temporary event-time residential systems into permanent urban communities. Using a Straussian grounded theory approach, the study analyzed six Olympic events including the Summer Olympics of 2008, 2012, 2016, 2020, and 2024 and the Winter Olympics of 2022. Using systematic coding across the six case studies, the study proposed the P-R-L model to explain the dynamic mechanisms through which Olympic Villages transform from temporary event spaces into long-term urban communities. The findings indicate that this transformation is not a linear process but rather a complex multi-dimensional structural evolution involving continuous interaction among adaptive infrastructure systems, governance arrangements, and long-term legacy production. The Physical dimension represents the material and organizational base for the transformation of the Olympic Village through its modular design, flexible use of space, and cooperative delivery methods. The Relational dimension facilitates changes in government policies and practices regarding community engagement and inclusion through dual-scenario governance systems, emergency management structures, and service network activation. The Legacy dimension consolidates long-term urban value through spatial integration, economic redevelopment, governance continuity, and resilience accumulation. The study emphasizes that the three dimensions coexist and interact throughout the entire Olympic Village lifecycle rather than operating sequentially. Its physical aspects can shape governance possibilities. Governance systems can affect legacy realization whereas long-term goals can significantly impact planning and operational decisions starting early in the development stage. Therefore, the proposed P-R-L framework offers a dynamic analytical perspective that differs from previous linear approaches to Olympic legacy research. The study adds to a larger body of literature related to temporary communities, mega-event governance, and urban resilience. By conceptualizing Olympic Villages as dynamic socio-spatial governance systems rather than temporary event-based facilities alone, the study emphasizes the need for governance continuity, institutional flexibility, and social integration to achieve sustainable post-Games transformation. Further, it provides a new understanding of Olympic legacy that recognizes intangible outcomes including governance resilience, institutional learning, and smart-city capabilities along with tangible outcomes such as physical redevelopment and economic growth. From a practical perspective, the study finds that fully integrating adaptive design of infrastructure, long-term coordinated governance strategies, and post-Games urban integration into planning for an Olympic Village from its inception can contribute to its successful transformation.

Despite these contributions, there are certain shortcomings in the study. First, because the long-term post-Games legacy of Paris 2024 is still evolving, the analysis is necessarily based on currently available planning documents, governance reports, sustainability strategies, and early implementation evidence rather than fully realized long-term legacy outcomes. Future longitudinal research should reassess the P-R-L framework as additional post-Games evidence becomes available. Second, the study primarily relies on documentary analysis. Documentation can be biased by the institutions providing it, and the quality of reporting varies across cases. Further research could include interviews with planners, administrators, community stakeholders, and post-Games residents to gain a greater understanding of governance dynamics and community transition processes. Moreover, although the use of a comparative multi-case methodology increases analytical generalizability, this study remains largely focused on the context of Olympic Villages. Future research could broaden application of the P-R-L framework to other forms of temporary urban systems such as international expos, emergency settlements, festival cities, or large-scale sporting event infrastructures. This study supports the argument that Olympic Villages are not merely temporary event-based infrastructures but evolving urban governance systems whose long-term success depends on the coordinated integration of spatial adaptability, relational governance, and legacy integration across multiple lifecycle phases.

## Supporting information

S1 File This file contains documents list for the analysis. (XLSX)

S2 File This file contains coding framework and representative evidence. (XLSX)
